# The longitudinal role of sleep on self‐harm during adolescence: A birth cohort study

**DOI:** 10.1111/jcpp.70018

**Published:** 2025-08-04

**Authors:** Michaela Pawley, Isabel Morales‐Muñoz, Andrew P Bagshaw, Nicole K Y Tang

**Affiliations:** ^1^ Department of Psychology University of Warwick Coventry UK; ^2^ School of Psychology University of Birmingham Birmingham UK; ^3^ Institute for Mental Health University of Birmingham Birmingham UK; ^4^ Centre for Human Brain Health University of Birmingham Birmingham UK

**Keywords:** Sleep, adolescence, longitudinal studies, self‐harm

## Abstract

**Background:**

Sleep problems and self‐harm during adolescence are both highly common and major public health concerns, yet the nature of their relationship remains poorly understood. This study examined the cross‐sectional and long‐term effects of several sleep phenotypes on self‐harm and whether decision‐making moderated this relationship.

**Methods:**

Data was utilised from the Millennium Cohort Study (*n* = 10,477, Female = 5,314 [50.72%]) when individuals were approximately 9 months, 14 years and 17 years of age. Sleep variables available were measured at 14 years and included sleep duration on weekdays and weekends, social jetlag, sleep onset latency and night awakenings. Self‐harm was measured at 14 and 17 years. The Cambridge Gambling Task assessed rational decision‐making at 14 years.

**Results:**

Cross‐sectionally, shorter sleep duration on school days (AOR = 0.875; 95% CI = 0.820, 0.933; *p* < .001), longer sleep onset latency (AOR = 1.005; 95% CI = 1.002, 1.007; *p* < .001) and more frequent night awakenings (AOR = 1.140; 95% CI = 1.086, 1.197; *p* < .001) were significantly associated with self‐harm at 14, even when controlling for demographic and clinical covariates. Longitudinal results indicated that shorter sleep duration on school days (AOR = 0.926; 95% CI = 0.874–0.982; *p* = .010), longer sleep onset latency (AOR = 1.003; 95% CI = 1.001–1.005; *p* = .008) and more frequent night awakenings (AOR = 1.090; 95% CI = 1.043–1.139; *p* < .001) also had a direct prospective effect on self‐harm at 17 when controlling for demographic and clinical factors. Rational decision‐making as measured by the Cambridge Gambling Task did not significantly contribute to this relationship.

**Conclusions:**

These findings highlight the prospective association between short sleep duration, increased sleep onset latency, fragmented sleep and self‐harm during adolescence. Ensuring adolescents obtain enough good quality, uninterrupted sleep appears critical to prevent engagement with self‐harm.

Self‐harm amongst adolescents is a major public health concern, with an estimated 14%–21% global prevalence (Lucena, Rossi, Azevedo, & Pereira, 2022) which has increased compared with previous years (Cybulski et al., 2021). Self‐harm may be conceptualised as the deliberate infliction of self‐injury or self‐poisoning regardless of intention to die and is a major risk factor for suicide (Hawton, Saunders, & O'Connor, 2012). Research has identified trends in the developmental trajectory of self‐harm, whereby behaviour typically begins prior to 12 years, peaks at around 14–16 years and decreases post 18 years of age (Geoffroy et al., 2022), highlighting adolescence as a critical period of vulnerability and prevention. Therefore, research has strived to investigate key risk factors for self‐harm, with sleep emerging as a potential feature of interest.

A global reduction in total sleep time and poor sleep patterns amongst older adolescents has been observed (Gradisar, Gardner, & Dohnt, 2011; Patte, Qian, & Leatherdale, 2017), which is concerning given sleep's critical role in regulating basic affective processes (Ben Simon, Vallat, Barnes, & Walker, 2020), which can influence self‐harm behaviour (Brereton & McGlinchey, 2020). Recent systematic reviews have validated the associations between sleep problems and self‐harm (Khazaie et al., 2021; Russell et al., 2019). For instance, short sleep duration (Hysing, Sivertsen, Stormark, & O'Connor, 2015), sleep disturbances (Asarnow et al., 2020; Nguyen et al., 2023), poor sleep quality (Asarnow et al., 2020; Liu, Chen, Bo, Fan, & Jia, 2017) and nightmares (Liu et al., 2017) are related to self‐harm during adolescence. Amongst adolescents, several sleep parameters influence self‐harm cross‐sectionally (Hysing et al., 2015; Kang et al., 2014; McGlinchey, Courtney‐Seidler, German, & Miller, 2017), and some recent evidence supports sleep being a potential precursor for later self‐harm (Fang et al., 2022; Liu, Tein, Jia, & Liu, 2021), yet the underlying mechanisms contributing to this relationship remains unclear.

Insufficient sleep influences cognitive functioning, including decision‐making (Satterfield & Killgore, 2019), which entails the selection of behaviour within a context involving risk that may be associated with both gains and losses (Buelow, Jungers, Parks, & Rinato, 2022). Brain maturation during adolescence enables strengthening of communication between the hippocampus and frontal lobe, facilitating the integration of memory and experience into rational decision‐making (Griffin, 2017). Particularly, ventromedial prefrontal cortex maturation is critical to the development of reward‐ and value‐based decision‐making (Hiser & Koenigs, 2018), a brain region heavily affected by sleep (Wang, Dai, Shao, Wang, & Zhou, 2022). Adolescents demonstrate riskier decision‐making compared with adults (Defoe, Semon Dubas, & Romer, 2019), frequently report sleep problems (Bruce, Lunt, & McDonagh, 2017) and rates of self‐harm engagement are increasing (Cybulski et al., 2021). Adolescents who engage in self‐harm also tend to exhibit risky decision‐making (McHugh et al., 2019; Oldershaw et al., 2009). Within the context of self‐harm, this may be related to the belief in attaining the quick reward of emotional relief, neglecting consideration of the potential long‐term negative outcomes (e.g. permanent damage; Lutz et al., 2022). Recency of adolescents' self‐injury episode has been positively associated with risky decision‐making (Oldershaw et al., 2009). Nevertheless, the temporal direction of associations that contribute to self‐harm remains unclear; therefore, longitudinal research is needed to establish the temporal precedence of decision‐making and self‐harm, to better understand the risk and potential protective factors contributing to these damaging behaviours.

The present study aims to investigate the cross‐sectional and longitudinal impact of several sleep parameters on self‐harm and the contributing role of decision‐making in this relationship during adolescence. While the cross‐sectional associations between poor sleep and self‐harm have been previously investigated, many studies have focused on a narrow range of sleep phenotypes and this study seeks to expand the range of sleep problems investigated. Furthermore, given the developmental course of self‐harm dominating adolescence (Geoffroy et al., 2022), and the lack of longitudinal studies, studying the prevalence and contributing factors at this critical age is clinically relevant. The main objectives of this study are to (a) explore cross‐sectional relationships between sleep duration, social jetlag, sleep onset latency and frequency of night awakenings on self‐harm; (b) investigate whether these aspects of sleep prospectively predict later self‐harm; and (c) explore whether rational decision‐making contributes to the relationship between sleep and later self‐harm. Based on previous studies (Asarnow et al., 2020; Hysing et al., 2015; Kang et al., 2014; Nguyen et al., 2023), we hypothesised that self‐reported shorter sleep duration, greater social jetlag, longer sleep onset latency and more frequent night awakenings would be related to self‐harm both cross‐sectionally and longitudinally. Furthermore, rational decision‐making would play a moderating role within this relationship.

## Methods

### Participants

The Millennium Cohort Study (MCS) is an ongoing multi‐disciplinary longitudinal birth cohort study that has been following young people born across the United Kingdom between 2000 and 2002. Participating families were sampled using a stratified cluster sampling design to oversample young people living in disadvantaged areas (Connelly & Platt, 2014). Seven sweeps of information have so far been collected in relation to parenting, general health, mental health, education and career aspirations, amongst other areas, with data collected from cohort members themselves, parents/caregivers, teachers and siblings. These sweeps have occurred approximately at the following time points (in relation to young people's age): 9 months, 3, 5, 7, 11, 14 and 17 years.

The current study utilises data from the sixth (14 years) and seventh (17 years) sweeps, with additional information being extracted from the first sweep (9 months) to account for covariate variables. The first sweep (9 months) of data collection obtained a sample of 18,818; the sixth wave (14 years) took place in 2015 and sampled 11,872 participants; and finally, the seventh sweep (17 years) sampled 10,757 participants in 2018. Only the first child, or cohort member, surveyed in the household was included in the sample, which aligns with other studies utilising the MCS (Lewis et al., 2022). Additional cohort members from the same household (i.e. twins, triplets) all utilised the same identification number which had implications on the clarity of results; thus, these cases were removed from the study. The resulting sample size for this study was 10,477 participants.

The MCS was approved by the UK National Health Service Research Ethics Committee, and written consent was received from all participating parents at each survey until the cohort members were of age to give their own consent (Shepherd & Gilbert, 2019). Data were downloaded from the UK Data Service, University of Essex, in December 2022, after registering with the UK Data Service before downloading. All the data for this study were publicly available.

### Measures

#### Measures of self‐reported sleep variables

The specific items relating to sleep were developed by the MCS team (Shepherd & Gilbert, 2019) and included six questions assessed at sweep six (14 years). These were converted to provide five measures of sleep for this study: sleep duration during school time, sleep duration during non‐school time, social jetlag, sleep onset latency and night awakenings. We note that some of these specific items have been assessed within validated sleep questionnaires, and further details on each of the sleep variables are provided in Appendix S1.

##### Sleep duration

Participants were asked what time they went to sleep and woke up on school and non‐school days. Response choices were recoded to reflect the approximate midpoint (e.g. ‘9–9:59 pm’ to ‘9:30 pm’) following prior research (Hisler, Twenge, & Krizan, 2020; Jackson & Testa, 2022). Sleep duration was then calculated based on the difference between bedtime and wake‐up time for school and non‐school nights to create two sleep duration variables (i.e. one for school and another for non‐school nights).

##### Social jetlag

Social jetlag (SJL) represents a misalignment between one's circadian and social clock. This was computed as the difference between the sleep midpoint on non‐school/free days (MSF) and school days (MSW) using Wittman's (Wittmann, Dinich, Merrow, & Roenneberg, 2006) original formula: SJL = [MSF – MSW].

##### Sleep onset latency

Participants were asked ‘During the last four weeks, how long did it usually take for you to fall asleep’. Mirroring previous research (Hisler et al., 2020), categorical responses were recoded as the midpoint (e.g. ‘16–30 min’ to ‘23 min’).

##### Night awakenings

The frequency of night awakenings was measured with the item ‘During the last four weeks, how often did you awaken during your sleep time and have trouble falling back to sleep again?’. Response options ranged from ‘All of the time’ to ‘None of the time’.

#### Outcome measure of self‐harm

Cohort members were asked about self‐harm at sweeps six (14 years) and seven (17 years) through self‐completion questionnaires. At sweep six, the following item was asked: ‘In the past year have you hurt yourself on purpose in any way?’ where responses were binary coded as ‘Yes’ (1) and ‘No’ (0). At sweep seven, cohort members answered the item ‘During the last year, have you hurt yourself on purpose in any of the following ways:’, with the following options displayed: ‘Cut or stabbed yourself’, ‘Burned yourself’, ‘Bruised or pinched yourself’, ‘Taken an overdose of tablets’, ‘Pulled out your hair’ and ‘Hurt yourself in some other way’. Responses were binary coded (‘Yes’ = 1, ‘No’ = 0). For this analysis, a new variable was created to indicate whether the participant engaged with any of the diverse methods of self‐harm whereby ‘1’ represented engagement with at least one of the responses and ‘0’ signalled complete non‐engagement.

#### Measure of decision‐making

The Cambridge Gambling Task (CGT; Rogers, 1999) assesses decision‐making and risk‐taking behaviour outside a learning context in both clinical and healthy populations (Romeu, Haines, Ahn, Busemeyer, & Vassileva, 2020). Further details of this specific task appear in Appendix S1.

This study focused exclusively on rational decision‐making, which measures the proportion of trials where the participant bet on the most likely outcome (a value of 1 reflects all trials involving the selection of the most likely colour). This dependent variable was chosen based on representing participants' tendency to make the optimum decision, given the explicit payoff and probability for each colour choice, thereby representing the ability to make rational decisions under risk. Poor rational decision‐making assessed with this measure has been associated with adolescent lifetime and repetitive nonsuicidal self‐injury (Lutz et al., 2022) and suicidality among young people (Chamberlain, Odlaug, Schreiber, & Grant, 2013).

#### Covariates

Several sociodemographic variables taken at 9 months were included in the analysis due to their likely influence on self‐harm, which were sex (female/male), family income (Below/Above Organisation for Economic Co‐operation and Development [OECD] 60% median poverty indicator) and ethnicity (white/other). The MCS provides information on 11 ethnic classification categories (see Appendix S1); however, for the purposes of this study, the sample was divided into white and non‐white participants due to the vast majority (i.e. 82.5%) being of white ethnicity. These covariates were selected based on prior research utilising the MCS data (Jackson & Testa, 2022; Scott, Biello, & Woods, 2019) as well as evidence supporting their influence on self‐harm‐related outcomes. Studies have shown that rates of self‐harm are typically highest among females (Hawton & Harriss, 2008), white adolescents (Farooq et al., 2021) and those who are from a lower socioeconomic background (Pitkänen, Bijlsma, Remes, Aaltonen, & Martikainen, 2021). Additionally, other covariates that were included in both the cross‐sectional and longitudinal analysis were self‐esteem, depressive symptoms and regular smoking at 14 years due to their associations with self‐harm (Moller, Tait, & Byrne, 2013; Oktan, 2017). Self‐esteem was measured using a shortened six‐item version of the Rosenberg self‐esteem scale (Rosenberg, 1965), with higher total scores indicating higher self‐esteem. Depressive symptoms were assessed using the Short Moods and Feelings Questionnaire (Angold et al., 1995). This questionnaire measures symptoms of depression experienced in the past fortnight, with higher values reflecting greater severity. Current smoking was measured with participants identifying as smoking at least one cigarette per week, following previous research (Laverty et al., 2019). Finally, self‐harm at 14 was controlled for when self‐harm at 17 was the outcome.

### Statistical analysis

Statistical analysis was performed using IBM SPSS Statistics (Version 29). First, to test for multicollinearity, all categorical predictors were transformed into sets of dummy variables and, along with the other predictor variables, run through a collinearity diagnostic in a regression model. All variables had a variance inflation factor below 2.5, warranting no further investigation into multicollinearity amongst predictors (Midi, Sarkar, & Rana, 2010). Second, descriptive statistics were calculated to obtain the means, standard deviations, frequencies and percentages of all variables of interest. Third, Pearson correlations were computed between all sleep predictor variables with rational decision‐making and linear covariates (depressive symptoms and self‐esteem at 14 years) to explore the initial associations between predictors and covariates. Fourth, multivariate logistic regression analyses were used to examine the associations of all the sleep measures in the same model with self‐harm cross‐sectionally and longitudinally. For the cross‐sectional analysis, an unadjusted model was computed (Model A), then an adjustment model (Model B). For Model B, sex, ethnicity, family income, depression, self‐esteem and currently smoking were additionally included. The longitudinal analysis used the same variables as the cross‐sectional analysis for Model A and B; however, previous self‐harm at 14 years was additionally included in the adjusted model. Odds ratios (ORs), adjusted ORs and 95% confidence intervals (CIs) were computed.

Finally, to test the potential contributing role of rational decision‐making at 14 years of age in the longitudinal associations between sleep and self‐harm, interaction models were tested. This interaction was tested with the sleep variables that were significantly associated with self‐harm at 17 years in Model B. The regression analyses included the interaction term as the predictor variable and mirrored the covariates utilised in the longitudinal analysis: (1) an unadjusted model and (2) an adjusted model.

#### Missing data

As 44.8% of participants from the original sample had dropped out at 17 years, several logistic regressions were conducted to identify significant factors related to attrition as part of the missing data pattern analysis to determine whether data were missing at random (see Table S1 in Appendix S1 for further details). In line with Seaman and White's (2013) review, response variables were selected based on potentially being confounding or prognostic variables. Furthermore, these were predominantly sociodemographic in nature due to their utility in improving external validity in cohort studies (Bonander, Nilsson, Björk, Bergström, & Strömberg, 2019). Based on the variables associated with selective dropout as the factors, a logistic regression model (response vs. nonresponse outcome) was created to establish propensity scores for each participant based on the conditional probability of participating at 17 years. The inverse of the propensity score was subsequently multiplied by the marginal probability of participating at 17 years to provide stabilised inverse probability weights. This method helps manage biases relating to attrition, limit type I errors and produce appropriate estimations of the variation of main effects (Pezzi, Cavo, Biggeri, Zamagni, & Nanni, 2016).

## Results

Table 1 provides the frequency and descriptive values of the sociodemographic, clinical, rational decision‐making and sleep variables of interest.

**Table 1:** Participant characteristics

	Baseline (9 months) (*N* = 18,539)			Sweep six (14 years) (*N* = 11,717)			Sweep seven (17 years) (*N* = 10,201)		
Sociodemographic characteristics									
	*N*	Frequency	%	*N*	Frequency	%	*N*	Frequency	%
Sex (Male/Female)	18,539	9526/9013	51.4/48.6	11,717	5879/5838	50.2/49.8	10,201	4995/5206	49.0/51.0
Ethnicity (White/Other)	18,492	15,277/3215	82.6/17.4	11,267	8964/2303	79.6/20.4	10,172	8228/1944	80.9/19.1
Family income (Above 60%/Below 60%)	18,458	11,602/6856	62.9/37.1	11,705	8245/3460	70.4/29.6	10,175	6965/3210	68.5/31.5

	*N*	Mean	*SD*	*N*	Mean	*SD*	*N*	Mean	*SD*
Birth weight, in kg	18,475	3.34	5.89	NA			NA		

Other variables									
	*N*	Frequency	%	*N*	Frequency	%	*N*	Frequency	%
Self‐harm (Yes/No)	NA			11,145	1634/9511	14.7/85.3	9,631	2203/7428	22.9/77.1
Currently smoking (Yes/No)	NA			11,146	218/10928	2.0/98.0	9,943	1120/8823	11.3/88.7

	*N*	Mean	*SD*	*N*	Mean	*SD*	*N*	Mean	*SD*
Self‐esteem (Score range 0 to 15)	NA			11,012	10.56	2.88	9,947	10.02	3.20
Depressive symptoms (Score range 0 to 26)	NA			11,045	5.52	5.85	NA		

Sleep and rational decision‐making variables									
	*N*	Mean	*SD*	*N*	Mean	*SD*	*N*	Mean	*SD*
Sleep duration, school day (Score range 5 to 13 hr)	NA			11,319	8.62	1.04	NA		
Sleep duration, non‐school day (Score range 7 to 16 hr)	NA			11,312	10.53	1.23	NA		
Social Jetlag (Score range −2 to 5.5 hr)	NA			11,300	1.97	0.83	NA		
Sleep onset latency (Score range 7.5 to 90 min)	NA			11,266	29.08	24.29	NA		
Night awakenings (Score range 0 to 5)	NA			11,302	1.38	1.37	NA		
Rational decision‐making (Score range 0 to 1)	NA			10,710	0.88	0.13	NA		

Correlation analyses revealed two moderate positive relations (*r* > 0.3) between both sleep onset latency and night awakenings with depressive symptoms at 14 years (*r* = .301; *p* < .001 and *r* = 0.377; *p* < .001, respectively; see Table S2 in Appendix S1). All other significant correlations were within the low range (*r* < 0.3), suggesting a weak association between variables.

### Sleep and self‐harm at 14 years: Cross‐sectional associations

The results from the cross‐sectional logistic regressions between sleep duration on school nights and non‐school nights, social jetlag, sleep onset time and night awakenings with self‐harm at 14 years old as an outcome are available in Table 2.

**Table 2:** Weighted unadjusted and adjusted associations between sleep variables at 14 and self‐harm at 14 years of age

	Model A (*N* = 10,477)			Model B (*N* = 10,233)		
	*β*	*p* Value	OR (95% CI)	*β*	*p* Value	OR (95% CI)
Sleep duration school day	−0.32	<.001	0.73 (0.69, 0.77)	−0.13	<.001	0.88 (0.82, 0.93)
Sleep duration non‐school day	−0.04	.103	0.96 (0.91, 1.01)	−0.04	.239	0.96 (0.91, 1.03)
Social jetlag	0.07	.070	1.08 (0.99, 1.16)	0.05	.272	1.05 (0.96, 1.16)
Sleep onset latency	0.01	<.001	1.01 (1.01, 1.01)	0.01	<.001	1.01 (1.00, 1.01)
Night awakenings	0.36	<.001	1.44 (1.38, 1.49)	0.13	<.001	1.14 (1.09, 1.20)
Sex	–	–	–	−0.48	<.001	0.62 (0.54, 0.72)
Family income	–	–	–	−0.01	.937	0.99 (0.87, 1.14)
Ethnicity	–	–	–	0.38	<.001	1.46 (1.19, 1.79)
Currently smoking	–	–	–	1.06	<.001	2.89 (2.04, 4.09)
Self‐esteem	–	–	–	−0.08	<.001	0.92 (0.90, 0.95)
Depressive symptoms	–	–	–	0.17	<.001	1.19 (1.17, 1.20)

In the unadjusted model (Model A), shorter sleep duration on school days (OR = 0.726; 95% CI = 0.687–0.767; *p* < .001), longer sleep onset time (OR = 1.011; 95% CI = 1.009–1.013; *p* < .001) and more frequent night awakenings (OR = 1.436; 95% CI = 1.382–1.492; *p* < .001) all demonstrated a significant relationship with self‐harm at 14 years.

Sleep duration on school days (OR = 0.875; 95% CI = 0.820–0.933; *p* < .001), sleep onset latency (OR = 1.005; 95% CI = 1.002–1.007; *p* < .001) and night awakenings (OR = 1.140; 95% CI = 1.086–1.197; *p* < .001) continued demonstrating significance in the same direction in the adjusted Model B.

### Sleep at 14 years and self‐harm at 17 years: Longitudinal associations

The longitudinal associations from the logistic regressions between all sleep variables at 14 years and self‐harm at 17 appear in Table 3.

**Table 3:** Weighted unadjusted and adjusted associations between sleep variables at 14 and self‐harm at 17 years of age

	Model A (*N* = 8,366)			Model B (*N* = 8,095)		
	*β*	*p* Value	OR (95% CI)	*β*	*p* Value	OR (95% CI)
Sleep duration school day	−0.20	<.001	0.82 (0.78, 0.87)	−0.08	.010	0.93 (0.87, 0.98)
Sleep duration non‐school day	−0.00	.932	1.00 (0.95, 1.05)	0.01	.813	1.01 (0.95, 1.06)
Social jetlag	−0.04	.227	0.96 (0.89, 1.03)	−0.07	.094	0.94 (0.86, 1.01)
Sleep onset latency	0.01	<.001	1.01 (1.01, 1.01)	0.00	.008	1.00 (1.00, 1.01)
Night awakenings	0.24	<.001	1.27 (1.22, 1.31)	0.09	<.001	1.09 (1.04, 1.14)
Sex	–	–	–	−0.25	<.001	0.78 (0.70, 0.88)
Family income	–	–	–	0.03	.621	1.03 (0.91, 1.17)
Ethnicity	–	–	–	0.39	<.001	1.48 (1.26, 1.75)
Regular smoking, 14 years	–	–	–	−0.02	.920	0.98 (0.67, 1.44)
Previous self‐harm, 14 years	–	–	–	0.88	<.001	2.40 (2.06, 2.80)
Self‐esteem, 14 years	–	–	–	−0.04	<.001	0.96 (0.94, 0.98)
Depressive symptoms, 14 years	–	–	–	0.06	<.001	1.06 (1.05, 1.07)

In Model A, decreased sleep duration on school days (OR = 0.822; 95% CI = 0.780–0.866; *p* < .001), longer sleep onset time (OR = 1.007; 95% CI = 1.005–1.009; *p* < .001) and more frequent night awakenings (OR = 1.266; 95% CI = 1.219–1.314; *p* < .001) were significantly associated with an increased likelihood of self‐harm at 17 years of age.

In Model B where several covariates were entered in the regression model, shorter sleep duration on school days (OR = 0.926; 95% CI = 0.874–0.982; *p* = .010), longer sleep onset latency (OR = 1.003; 95% CI = 1.001–1.005; *p* = .008) and more frequent night awakenings remained significant predictors for self‐harm at 17 (OR = 1.090; 95% CI = 1.043–1.139; *p* < .001).

### Interaction analyses: The moderating role of decision‐making between sleep and self‐harm

Sleep variables that were significantly associated with later self‐harm at 17 years in Model B (i.e. sleep duration on school days, sleep onset latency and night awakenings) were used in the interaction models with rational decision‐making (see Table 4). Sleep duration on school days (OR = 0.688; 95% CI = 0.478–0.991; *p* = .044) and sleep onset latency (OR = 1.018; 95% CI = 1.003–1.033; *p* = .021) had a significant interaction with rational decision‐making in the unadjusted models, while night awakenings were not significant in the interaction (OR = 1.243; 95% CI = 0.949–1.626; *p* = .114). No significant interactions were found in any of the adjusted models. In all interaction models, being female, white, prior self‐harm, having low self‐esteem and high levels of depressive symptoms were significantly associated with later self‐harm (see Appendix S1: Table S3).

**Table 4:** Weighted unadjusted and adjusted interactions between sleep and rational decision‐making at 14 years with self‐harm at 17 years

	Model A			Model B		
	*β*	*p* Value	OR (95% CI)	*β*	*p* Value	OR (95% CI)
Sleep duration school day × RDM	−0.37	.044	0.69 (0.48, 0.99)	−0.26	.223	0.77 (0.51, 1.17)
Sleep duration school day	0.06	.732	1.06 (0.77, 1.46)	0.14	.465	1.15 (0.79, 1.66)
RDM	3.23	.043	25.24 (1.11, 572.13)	2.27	.212	9.68 (0.27, 343.53)
Sleep onset latency × RDM	0.02	.021	1.02 (1.00, 1.03)	0.01	.221	1.01 (0.99, 1.03)
Sleep onset latency	−0.00	.629	1.00 (0.98, 1.01)	−0.01	.503	1.00 (0.98, 1.01)
RDM	−0.56	.079	0.57 (0.30, 1.07)	−0.29	.415	0.75 (0.38, 1.50)
Night awakenings × RDM	0.22	.114	1.24 (0.95, 1.63)	0.14	.385	1.15 (0.84, 1.56)
Night awakenings	0.10	.398	1.11 (0.87, 1.41)	−0.01	.940	0.99 (0.75, 1.30)
RDM	−0.17	.587	0.84 (0.46, 1.56)	−0.12	.718	0.89 (0.46, 1.72)

RDM, rational decision‐making.

### Post‐hoc mediation analysis: The mediating role of decision‐making between sleep and self‐harm

In addition to the primary analyses, we conducted an exploratory post‐hoc analysis to further investigate the role of decision‐making as a mediator in the association between sleep variables that were significant predictors for longitudinal self‐harm (see Appendix S1). The mediation analyses did not reveal any significant indirect effects.

## Discussion

This study aimed to shed light on the impact of several sleep parameters (sleep duration on school and non‐school nights, social jetlag, sleep onset latency and night awakenings) on self‐harm, and the contributing role of decision‐making in this relationship during adolescence using a population‐based birth cohort from the UK. The findings complement the existing body of research, but importantly build upon it by identifying specific sleep phenotypes that are both cross‐sectionally and longitudinally associated with self‐harm during a critical developmental period.

Consistent with previous findings, adolescents who were experiencing poorer sleep were more likely to engage in self‐harm cross‐sectionally (McGlinchey et al., 2017). Specifically, shorter sleep duration on school days, longer sleep onset latency and more frequent night awakenings were associated with an increased likelihood of engaging in self‐harm at 14 years. When accounting for sociodemographic and clinical variables, these sleep variables remained significant predictors for self‐harm at 17 years in the same direction. These findings highlight specific sleep parameters as robust predictors for both concurrent and prospective self‐harm risk (Christenfeld, Sloan, Carroll, & Greenland, 2004; Fulfs, Poulain, Vogel, Nenoff, & Kiess, 2024; Su, John, & Lin, 2023).

### Sleep and prospective self‐harm risk

Night awakenings may be considered a unique predictor in our study due to its influence on both micro‐ and macro‐sleep architecture (Medrzycka‐Dabrowska, Lewandowska, Kwiecień‐Jaguś, & Czyż‐Szypenbajl, 2018). Interruptions in rapid eye movement (REM) sleep caused by night awakenings have been associated with compromised emotion regulation (Wassing et al., 2019). REM sleep plays a critical role in emotional processing (Trouche, Pompili, & Girardeau, 2020), and its deprivation has been linked with increased emotional reactivity (Rosales‐Lagarde et al., 2012). Individuals who awaken frequently throughout the night may struggle to obtain enough REM sleep (Vuong, York, Garrett, Connolly, & Devergnas, 2021), developing deficits in emotion regulation and one's ability to implement healthy emotional responses (Riemann et al., 2012). This could be one potential explanation for the unique association between fragmented sleep and later self‐harm in our study.

Research exploring arousal and coping skills may further elucidate the mechanisms underlying the relationship between sleep fragmentation and self‐harm. More specifically, individuals who report difficulty initiating and/or maintaining sleep tend to appraise their lives as more stressful and utilise emotion‐focused coping strategies (Morin, Rodrigue, & Ivers, 2003). Within the context of self‐harm, adolescents who struggle to maintain sleep during the night may be more likely to negatively appraise life events and respond using emotion‐oriented coping strategies (e.g. self‐harm) over directing efforts towards problem‐solving. Such deficits in problem‐solving have been observed in studies investigating the effects of insomnia on cognitive performance (Fortier‐Brochu, Beaulieu‐Bonneau, Ivers, & Morin, 2012; Wardle‐Pinkston, Slavish, & Taylor, 2019). Nevertheless, previous research tends to assess the effects of insomnia, rather than specific sleep markers. Our study observed differentiating strengths of the effect between frequency of night awakenings and sleep onset latency with prospective self‐harm, two key factors that constitute an insomnia diagnosis, highlighting the importance in investigating specific sleep markers with mental health outcomes.

The effects of insufficient sleep duration on increased likelihood of engaging with self‐harm amongst adolescents are well‐documented (Fang et al., 2022; Hysing et al., 2015; Kang et al., 2014). Our results indicated that shorter sleep duration during school days (or week/workdays) associates with concurrent and prospective engagement with self‐harm. Given the cumulative effect of short sleep duration during the week contributing to chronic sleep deprivation, and the effect that this has on mood, somatic and psychological symptomology (Seton & Fitzgerald, 2021), adolescents reporting shorter sleep duration during school days may be more likely to engage with self‐harm related behaviours.

Sleep onset latency was also significantly associated with self‐harm cross‐sectionally and longitudinally, which may be explained based on research on cognitive disengagement syndrome (CDS). CDS is an umbrella term for several attentional behaviours relating to slowed information processing and behaviour, mental confusion, hypoactivity and distractibility (Fredrick, Yeaman, Yu, Langberg, & Becker, 2022) and is often comorbid with ADHD (Burns & Becker, 2021). It has been associated with impairments in organisation and problem‐solving (Wood, Lewandowski, Lovett, & Antshel, 2017) and internalising functioning (Becker, Burns, Smith, & Langberg, 2020). Longer sleep onset time has demonstrated links with both ADHD (Hvolby, 2015) and CDS (Fredrick et al., 2022), and its symptoms of daydreaming and sluggishness have been shown to distinctively relate to various self‐harm functions amongst adolescents, beyond psychiatric symptoms (Torun et al., 2021). Broad attentional difficulties may partially account for the underlying mechanisms connecting longer sleep onset latency to self‐harm.

Shorter sleep duration on school days, longer sleep onset latency and more frequent night awakenings pose specific risk factors for engagement with self‐harm both cross‐sectionally and longitudinally. Further research is necessary to identify the underlying mechanisms and contributing factors that may account for particular sleep variables being more disruptive in relation to self‐harm across adolescence.

### The role of decision‐making

Adolescence is marked by a tendency towards poor decision‐making, as impulse control and response inhibition systems compete with a hyper‐responsiveness to rewards, particularly in situations that involve emotion and social factors (Blakemore & Robbins, 2012). Poor sleep can negatively influence decision‐making capacity (Venkatraman, Chuah, Huettel, & Chee, 2007) and adolescents who engage in self‐harm demonstrate poor decision‐making (McHugh et al., 2019; Oldershaw et al., 2009). Despite this, our results indicated that rational decision‐making did not significantly contribute to or underlie the association between sleep and later self‐harm.

Ackerman et al. (2015) similarly found adolescents with a history of attempting suicide to display an equivalent capacity to make rational choices compared with healthy controls, although differences were observed in risk‐taking propensity through a riskier average betting ratio. This was attributed to participants minimising potential aversive outcomes within a context of uncertainty (Ackerman et al., 2015). Despite adolescents' ability to make rational decisions, they may be more likely to engage in behaviour that leads to attaining faster rewards (e.g. self‐harming to experience emotional relief) over long‐term natural rewards, which can contribute to increased resilience and improved mental health (Geschwind et al., 2010). This better reflects a risk‐averse decision‐making strategy, while our study is limited to examining adolescents' overall rational decision‐making ability. Future research should consider investigating additional decision‐making subtypes, such as risk adjustment or impulsivity via neurocognitive tasks such as the CGT (Rogers, 1999) or Iowa Gambling Task (Bechara, Damasio, Damasio, & Anderson, 1994), to better determine the relationship between sleep and this aspect of executive function with self‐harm.

### Strengths and limitations

This study has several strengths, including the longitudinal setting using a population‐based sample, utilising multiple measurement points for self‐harm that cover long‐term engagement, incorporating a neurocognitive task to quantify decision‐making performance and investigating multiple sleep parameters. However, there are some limitations which are typical among large cohort studies. First, the sleep variables that were investigated in this study were self‐reported, which inflates the possibility of responder bias. For example, greater sleep problems are subjectively reported compared with objective measures among youths with depression (Bertocci et al., 2005) and anxiety (Alfano, Patriquin, & De Los Reyes, 2015). Nevertheless, sleep is a multidimensional construct with significant individual variation (Fabbri et al., 2021); therefore, assessing several facets of sleep, including perception through subjective measures and consideration for factors that may influence this (e.g. mental health), is recommended to capture the complex nature of sleep. Additionally, while several sleep measures were assessed at 14 years, only one parameter was measured in the later sweep at 17 years, preventing a meaningful investigation of how self‐harm at 14 years might predict sleep problems at 17 years. Future research investigating the bidirectional associations between sleep and self‐harm utilising multiple sleep measures may validate the specificity of this relationship.

Second, some sleep measures captured a small window of variability and specificity in their response options. For example, bedtime response choices for both school and non‐school nights had a limited range of ‘Before 9 pm’ to ‘After midnight’, which is also ambiguous. Adolescents often display significant differences in bedtime between school and non‐school nights (Gradisar et al., 2011), including additional later bedtime options would provide more accurate measurements of sleep patterns on non‐school nights. Furthermore, by not specifying time asleep, bedtime could additionally capture both sleep onset latency and night awakenings. Moreover, assessing the validity of our sleep measures was not possible due to being single‐item measures that were not directly derived from an existing scale. Future research utilising validated sleep questionnaires for adolescents, such as the Adolescent Sleep Wake Scale (LeBourgeois, Giannotti, Cortesi, Wolfson, & Harsh, 2005), may help address potential measurement biases and strengthen the quality of research findings.

Third, this study is limited in offering insight to a general pathway of self‐harm, without identifying adolescents at greater risk of suicidal behaviours. Previous research has highlighted the differential purposes and functions of self‐injurious behaviour based on intent to die, and inconsistencies in terminology and relationship to outcomes have been argued as limiting progress in preventing the occurrence of self‐harm related behaviours (Clarke, Allerhand, & Berk, 2019). Future large‐scale studies should seek to specify the typology of self‐harm to provide more precise data.

Fourth, the items assessing sleep onset latency and frequency of night awakenings were ambiguous, with response options pertaining to the previous 4 weeks lacking a meaningful quantitative value. Previous research has been criticised for missing well‐defined criteria to assess night awakenings or having significant limitations in its assessment (Ohayon, Krystal, Roehrs, Roth, & Vitiello, 2010). Detail on sleep onset latency and the frequency of awakenings per night and week would provide greater clarification on the true impact of these sleep parameters on self‐harm.

Fifth, while the study is longitudinal, only two waves of data on sleep and self‐harm behaviour were available. Our models were adjusted by confounders of background factors and age 14 mental health variables and did not include intermediate variables as they may have resulted in overadjustment (Ananth & Schisterman, 2017); however, other unmeasured confounds may still have biased the observed effects. Future longitudinal studies should seek to expand the number of observations by incorporating data from additional sweeps to accurately map the trajectory of sleep problems and self‐harm engagement over adolescence. This will allow for investigation into other potential mediators, like psychosocial problems and psychological distress, as multiple data waves can establish the temporal ordering of effects and causation (Li, Yoshida, Kaufman, & Mathur, 2023). This may provide clinical insight through better understanding the long‐term effects of poor sleep on self‐harm and identification of critical underlying mechanisms.

Lastly, although we utilised stabilised inverse probability weighting (SIPW) to account for attrition and data missingness, previous work has suggested inverse probability weighting is not always effective in reducing selection bias when attrition results in missing outcomes (Metten, Costet, Multigner, Viel, & Chauvet, 2022). It is important to highlight SIPW can help address this bias (Pezzi et al., 2016); however, future studies may benefit from exploring whether alternative weighting methods, such as the Stable Estimand AdaptiVE IPW estimation (Avagyan & Vansteelandt, 2021), or multiple imputation, influence the robustness of findings. There is limited theoretical and empirical understanding into which weighting method is most sound (Schmidt & Woll, 2017), indicating that this area of methodology requires further investigation.

In conclusion, short sleep duration on school days, longer sleep onset latency and more frequent night awakenings are associated with self‐harm both cross‐sectionally at 14 years and longitudinally at 17 years. These findings highlight the prospective role of poor sleep on self‐harm‐related behaviours. Cognitive‐behavioural therapy for insomnia may address the sleep problems featured in this study (Riemann et al., 2023); however, further research is still needed to understand the manifestations and models of sleep problems within the adolescent population (Dewald‐Kaufmann, de Bruin, & Michael, 2019). Despite the development of various etiologic adult models of insomnia (Perlis, Shaw, Cano, & Espie, 2011), there is an absence of understanding the interplay of factors associated with adolescent sleep problems, which may influence the effectiveness of applying cognitive‐behavioural therapy for insomnia as a sleep intervention for this demographic (Meltzer, Wainer, Engstrom, Pepa, & Mindell, 2021). This study adds to existing research in sleep and self‐harm by highlighting the relevance of targeting sleep duration on school days, sleep latency and nighttime awakenings to prevent engagement with self‐harm‐related behaviours. Specifically, ensuring adolescents have sufficient, good quality, non‐fragmented sleep appears critical to prevent engagement with self‐harm during this critical developmental period.

## Ethical statement

The MCS was approved by the UK National Health Service Research Ethics Committee, and written consent was received from all participating parents at each survey until the cohort members were of age to give their own consent (Shepherd & Gilbert, 2019).

**Key points**

- Sleep problems have been identified as a modifiable risk factor for self‐harm; however, the nature of this relationship remains poorly understood.
- The UK Millennium Cohort Study (MCS), a nationally representative longitudinal birth cohort dataset, has provided insight into the cross‐sectional and longitudinal effects of several sleep phenotypes on engagement with self‐harm during adolescence.
- Shorter sleep duration on school days, longer sleep onset latency and more frequent night awakenings at 14 years of age were significantly associated with self‐harm cross‐sectionally and prospectively at 17 years of age.
- Rational decision‐making was not shown to contribute to the relationship between sleep and longitudinal self‐harm; therefore, further work is needed to identify the underlying mechanisms that exacerbate the effects of sleep on self‐harm.

## Supporting information


**Appendix S1.** Supplementary material.
**Table S1.** Associations between various variables possibly related to attrition and participation at 17 years of age.
**Table S2.** Correlations between all sleep variables with rational decision‐making, depressive symptoms at 14, self‐esteem at 14 years.
**Table S3.** Weighted unadjusted and adjusted interactions between sleep and rational decision‐making at 14 years with self‐harm at 17 years.
**Figure S1.** Direct path diagram between sleep variables at 14 years, rational decision‐making at 14 years, and self‐harm at 17 years, with sex, ethnicity, prior self‐harm, self‐esteem, and depressive symptoms at 14 as control variables.

## Data Availability

The University of London Centre for Longitudinal Studies owns the copyright for the MCS data used in this study. The MCS data are held/curated by the UK Data Service. Anyone wishing to use the MCS data (found at: https://discover.ukdataservice.ac.uk/series/?sn=2000031) must register and submit a data request to the UK Data Service at http://ukdataservice.ac.uk/. Additional terms and conditions of access are outlined here: https://www.ukdataservice.ac.uk/get‐data/how‐to access/conditions.

## References

[jcpp70018-bib-0001] Ackerman, J.P. , McBee‐Strayer, S.M. , Mendoza, K. , Stevens, J. , Sheftall, A.H. , Campo, J.V. , & Bridge, J.A. (2015). Risk‐sensitive decision‐making deficit in adolescent suicide attempters. Journal of Child and Adolescent Psychopharmacology, 25, 109–113. 25265242 10.1089/cap.2014.0041 PMC4367520

[jcpp70018-bib-0092] Alfano, C.A. , Patriquin, M.A. , & De Los Reyes, A. (2015). Subjective – objective sleep comparisons and discrepancies among clinically‐anxious and healthy children. Journal of Abnormal Child Psychology, 43, 1343–1353. 25896729 10.1007/s10802-015-0018-7 PMC7364662

[jcpp70018-bib-0002] Ananth, C.V. , & Schisterman, E.F. (2017). Confounding, causality, and confusion: The role of intermediate variables in interpreting observational studies in obstetrics. American Journal of Obstetrics and Gynecology, 217, 167–175. 28427805 10.1016/j.ajog.2017.04.016 PMC5545051

[jcpp70018-bib-0003] Angold, A. , Costello, E.J. , Messer, S.C. , Pickles, A. , Winder, F. , & Silver, D. (1995). The development of a questionnaire for use in epidemiological studies of depression in children and adolescents. International Journal of Methods in Psychiatric Research, 5, 237–249.

[jcpp70018-bib-0004] Asarnow, J.R. , Bai, S. , Babeva, K.N. , Adrian, M. , Berk, M.S. , Asarnow, L.D. , … & McCauley, E. (2020). Sleep in youth with repeated self‐harm and high suicidality: Does sleep predict self‐harm risk? Suicide and Life‐threatening Behavior, 50, 1189–1197. 32706147 10.1111/sltb.12658 PMC9327783

[jcpp70018-bib-0005] Avagyan, V. , & Vansteelandt, S. (2021). Stable inverse probability weighting estimation for longitudinal studies. Scandinavian Journal of Statistics, 48, 1046–1067.

[jcpp70018-bib-0006] Bechara, A. , Damasio, A.R. , Damasio, H. , & Anderson, S.W. (1994). Insensitivity to future consequences following damage to human prefrontal cortex. Cognition, 50, 7–15. 8039375 10.1016/0010-0277(94)90018-3

[jcpp70018-bib-0007] Becker, S.P. , Burns, G.L. , Smith, Z.R. , & Langberg, J.M. (2020). Sluggish cognitive tempo in adolescents with and without ADHD: Differentiation from adolescent‐reported ADHD inattention and unique associations with internalizing domains. Journal of Abnormal Child Psychology, 48, 391–406. 31814060 10.1007/s10802-019-00603-9 PMC7007365

[jcpp70018-bib-0008] Ben Simon, E. , Vallat, R. , Barnes, C.M. , & Walker, M.P. (2020). Sleep loss and the socio‐emotional brain. Trends in Cognitive Sciences, 24, 435–450. 32299657 10.1016/j.tics.2020.02.003

[jcpp70018-bib-0091] Bertocci, M.A. , Dahl, R.E. , Williamson, D.E. , Iosif, A.‐M. , Birmaher, B. , Axelson, D. , & Ryan, N.D. (2005). Subjective sleep complaints in pediatric depression: A controlled study and comparison with EEG measures of sleep and waking. Journal of the American Academy of Child & Adolescent Psychiatry, 44, 1158–1166. 16239865 10.1097/01.chi.0000179057.54419.17

[jcpp70018-bib-0009] Blakemore, S.‐J. , & Robbins, T.W. (2012). Decision‐making in the adolescent brain. Nature Neuroscience, 15, 1184–1191. 22929913 10.1038/nn.3177

[jcpp70018-bib-0010] Bonander, C. , Nilsson, A. , Björk, J. , Bergström, G.M.L. , & Strömberg, U. (2019). Participation weighting based on sociodemographic register data improved external validity in a population‐based cohort study. Journal of Clinical Epidemiology, 108, 54–63. 30562543 10.1016/j.jclinepi.2018.12.011

[jcpp70018-bib-0011] Brereton, A. , & McGlinchey, E. (2020). Self‐harm, emotion regulation, and experiential avoidance: A systematic review. Archives of Suicide Research, 24(Suppl 1), 1–24. 10.1080/13811118.2018.1563575 30636566

[jcpp70018-bib-0012] Bruce, E.S. , Lunt, L. , & McDonagh, J.E. (2017). Sleep in adolescents and young adults. Clinical Medicine, 17, 424–428. 28974591 10.7861/clinmedicine.17-5-424 PMC6301929

[jcpp70018-bib-0013] Buelow, M.T. , Jungers, M.K. , Parks, C. , & Rinato, B. (2022). Contextual factors affecting risky decision making: The influence of music on task performance and perceived distraction. Frontiers in Psychology, 13, 818689. 35310222 10.3389/fpsyg.2022.818689 PMC8926386

[jcpp70018-bib-0014] Burns, G.L. , & Becker, S.P. (2021). Sluggish cognitive tempo and ADHD symptoms in a nationally representative sample of U.S. children: Differentiation using categorical and dimensional approaches. Journal of Clinical Child & Adolescent Psychology, 50, 267–280. 31671271 10.1080/15374416.2019.1678165 PMC7190416

[jcpp70018-bib-0015] Chamberlain, S.R. , Odlaug, B.L. , Schreiber, L.R.N. , & Grant, J.E. (2013). Clinical and neurocognitive markers of suicidality in young adults. Journal of Psychiatric Research, 47, 586–591. 23357208 10.1016/j.jpsychires.2012.12.016

[jcpp70018-bib-0016] Christenfeld, N.J.S. , Sloan, R.P. , Carroll, D. , & Greenland, S. (2004). Risk factors, confounding, and the illusion of statistical control. Psychosomatic Medicine, 66, 868–875. 15564351 10.1097/01.psy.0000140008.70959.41

[jcpp70018-bib-0017] Clarke, S. , Allerhand, L.A. , & Berk, M.S. (2019). Recent advances in understanding and managing self‐harm in adolescents. F1000Research, 8. 10.12688/f1000research.19868.1 PMC6816451 31681470

[jcpp70018-bib-0018] Connelly, R. , & Platt, L. (2014). Cohort profile: UK Millennium Cohort Study (MCS). International Journal of Epidemiology, 43, 1719–1725. 24550246 10.1093/ije/dyu001

[jcpp70018-bib-0019] Cybulski, L. , Ashcroft, D.M. , Carr, M.J. , Garg, S. , Chew‐Graham, C.A. , Kapur, N. , & Webb, R.T. (2021). Temporal trends in annual incidence rates for psychiatric disorders and self‐harm among children and adolescents in the UK, 2003–2018. BMC Psychiatry, 21, 229. 33941129 10.1186/s12888-021-03235-w PMC8092997

[jcpp70018-bib-0020] Defoe, I.N. , Semon Dubas, J. , & Romer, D. (2019). Heightened adolescent risk‐taking? Insights from lab studies on age differences in decision‐making. Policy Insights From the Behavioral and Brain Sciences, 6, 56–63.

[jcpp70018-bib-0021] Dewald‐Kaufmann, J. , de Bruin, E. , & Michael, G. (2019). Cognitive Behavioral Therapy for Insomnia (CBT‐i) in school‐aged children and adolescents. Sleep Medicine Clinics, 14, 155–165. 31029183 10.1016/j.jsmc.2019.02.002

[jcpp70018-bib-0022] Fabbri, M. , Beracci, A. , Martoni, M. , Meneo, D. , Tonetti, L. , & Natale, V. (2021). Measuring subjective sleep quality: A review. International Journal of Environmental Research and Public Health, 18, 1082. 33530453 10.3390/ijerph18031082 PMC7908437

[jcpp70018-bib-0023] Fang, J. , Wan, Y. , Zhang, X. , Su, P. , Tao, F. , & Sun, Y. (2022). Sleep duration trajectory during the transition to adolescence and subsequent risk of non‐suicidal self‐harm. European Child & Adolescent Psychiatry, 31, 1–9. 10.1007/s00787-021-01768-9 33825948

[jcpp70018-bib-0024] Farooq, B. , Clements, C. , Hawton, K. , Geulayov, G. , Casey, D. , Waters, K. , … & Kapur, N. (2021). Self‐harm in children and adolescents by ethnic group: An observational cohort study from the multicentre study of self‐harm in England. The Lancet Child & Adolescent Health, 5, 782–791. 34555352 10.1016/S2352-4642(21)00239-X PMC9766885

[jcpp70018-bib-0025] Fortier‐Brochu, É. , Beaulieu‐Bonneau, S. , Ivers, H. , & Morin, C.M. (2012). Insomnia and daytime cognitive performance: A meta‐analysis. Sleep Medicine Reviews, 16, 83–94. 21636297 10.1016/j.smrv.2011.03.008

[jcpp70018-bib-0026] Fredrick, J.W. , Yeaman, K.M. , Yu, X. , Langberg, J.M. , & Becker, S.P. (2022). A multi‐method examination of sluggish cognitive tempo in relation to adolescent sleep, daytime sleepiness, and circadian preference. Journal of Child Psychology and Psychiatry, 63, 1658–1667. 35045192 10.1111/jcpp.13568

[jcpp70018-bib-0027] Fulfs, T. , Poulain, T. , Vogel, M. , Nenoff, K. , & Kiess, W. (2024). Associations between sleep problems and emotional/behavioural difficulties in healthy children and adolescents. BMC Pediatrics, 24, 15. 38183087 10.1186/s12887-023-04487-z PMC10768421

[jcpp70018-bib-0028] Geoffroy, M.‐C. , Bouchard, S. , Per, M. , Khoury, B. , Chartrand, E. , Renaud, J. , … & Orri, M. (2022). Prevalence of suicidal ideation and self‐harm behaviours in children aged 12 years and younger: A systematic review and meta‐analysis. The Lancet Psychiatry, 9, 703–714. 35907406 10.1016/S2215-0366(22)00193-6

[jcpp70018-bib-0029] Geschwind, N. , Peeters, F. , Jacobs, N. , Delespaul, P. , Derom, C. , Thiery, E. , … & Wichers, M. (2010). Meeting risk with resilience: High daily life reward experience preserves mental health. Acta Psychiatrica Scandinavica, 122, 129–138. 20064128 10.1111/j.1600-0447.2009.01525.x

[jcpp70018-bib-0031] Gradisar, M. , Gardner, G. , & Dohnt, H. (2011). Recent worldwide sleep patterns and problems during adolescence: A review and meta‐analysis of age, region, and sleep. Sleep Medicine, 12, 110–118. 21257344 10.1016/j.sleep.2010.11.008

[jcpp70018-bib-0032] Griffin, A. (2017). Adolescent neurological development and implications for health and well‐being. Healthcare (Basel), 5, 62. 28961184 10.3390/healthcare5040062 PMC5746696

[jcpp70018-bib-0033] Hawton, K. , & Harriss, L. (2008). The changing gender ratio in occurrence of deliberate self‐harm across the lifecycle. Crisis, 29, 4–10. 18389640 10.1027/0227-5910.29.1.4

[jcpp70018-bib-0034] Hawton, K. , Saunders, K.E. , & O'Connor, R.C. (2012). Self‐harm and suicide in adolescents. The Lancet, 379, 2373–2382. 10.1016/S0140-6736(12)60322-5 22726518

[jcpp70018-bib-0035] Hiser, J. , & Koenigs, M. (2018). The multifaceted role of the ventromedial prefrontal cortex in emotion, decision making, social cognition, and psychopathology. Biological Psychiatry, 83, 638–647. 29275839 10.1016/j.biopsych.2017.10.030 PMC5862740

[jcpp70018-bib-0036] Hisler, G. , Twenge, J.M. , & Krizan, Z. (2020). Associations between screen time and short sleep duration among adolescents varies by media type: Evidence from a cohort study. Sleep Medicine, 66, 92–102. 31838456 10.1016/j.sleep.2019.08.007

[jcpp70018-bib-0037] Hvolby, A. (2015). Associations of sleep disturbance with ADHD: Implications for treatment. ADHD Attention Deficit and Hyperactivity Disorders, 7, 1–18. 10.1007/s12402-014-0151-0 PMC4340974 25127644

[jcpp70018-bib-0038] Hysing, M. , Sivertsen, B. , Stormark, K.M. , & O'Connor, R.C. (2015). Sleep problems and self‐harm in adolescence. British Journal of Psychiatry, 207, 306–312. 10.1192/bjp.bp.114.146514 26206862

[jcpp70018-bib-0039] Jackson, D.B. , & Testa, A. (2022). Police stops and adolescent sleep problems: Findings from the UK millennium cohort study. Journal of Sleep Research, 31, e13585. 35289002 10.1111/jsr.13585 PMC9786844

[jcpp70018-bib-0040] Kang, S.‐G. , Lee, Y.J. , Kim, S.J. , Lim, W. , Lee, H.‐J. , Park, Y.‐M. , … & Hong, J.P. (2014). Weekend catch‐up sleep is independently associated with suicide attempts and self‐injury in Korean adolescents. Comprehensive Psychiatry, 55, 319–325. 24267542 10.1016/j.comppsych.2013.08.023

[jcpp70018-bib-0042] Khazaie, H. , Zakiei, A. , McCall, W.V. , Noori, K. , Rostampour, M. , Sadeghi Bahmani, D. , & Brand, S. (2021). Relationship between sleep problems and self‐injury: A systematic review. Behavioral Sleep Medicine, 19, 689–704. 32991212 10.1080/15402002.2020.1822360

[jcpp70018-bib-0043] Laverty, A.A. , Filippidis, F.T. , Taylor‐Robinson, D. , Millett, C. , Bush, A. , & Hopkinson, N.S. (2019). Smoking uptake in UK children: Analysis of the UK Millennium Cohort Study. Thorax, 74, 607–610. 30442657 10.1136/thoraxjnl-2018-212254

[jcpp70018-bib-0044] LeBourgeois, M.K. , Giannotti, F. , Cortesi, F. , Wolfson, A.R. , & Harsh, J. (2005). The relationship between reported sleep quality and sleep hygiene in Italian and American adolescents. Pediatrics, 115(Suppl 1), 257–265. 15866860 10.1542/peds.2004-0815H PMC3928632

[jcpp70018-bib-0045] Lewis, G. , Srinivasan, R. , Roiser, J. , Blakemore, S.‐J. , Flouri, E. , & Lewis, G. (2022). Risk‐taking to obtain reward: Sex differences and associations with emotional and depressive symptoms in a nationally representative cohort of UK adolescents. Psychological Medicine, 52, 2805–2813. 33431091 10.1017/S0033291720005000 PMC9647510

[jcpp70018-bib-0046] Li, Y. , Yoshida, K. , Kaufman, J.S. , & Mathur, M.B. (2023). A brief primer on conducting regression‐based causal mediation analysis. Psychological Trauma Theory Research Practice and Policy, 15, 930–938. 36701540 10.1037/tra0001421 PMC10368791

[jcpp70018-bib-0047] Liu, X. , Chen, H. , Bo, Q.‐G. , Fan, F. , & Jia, C.‐X. (2017). Poor sleep quality and nightmares are associated with non‐suicidal self‐injury in adolescents. European Child & Adolescent Psychiatry, 26, 271–279. 27383464 10.1007/s00787-016-0885-7

[jcpp70018-bib-0048] Liu, Z.‐Z. , Tein, J.‐Y. , Jia, C.‐X. , & Liu, X. (2021). Depression as a mediator between frequent nightmares and non‐suicidal self‐injury among adolescents: A 3‐wave longitudinal model. Sleep Medicine, 77, 29–34. 33307303 10.1016/j.sleep.2020.11.015

[jcpp70018-bib-0049] Lucena, N.L. , Rossi, T.A. , Azevedo, L.M.G. , & Pereira, M. (2022). Self‐injury prevalence in adolescents: A global systematic review and meta‐analysis. Children and Youth Services Review, 142, 106634.

[jcpp70018-bib-0050] Lutz, N.M. , Chamberlain, S.R. , Goodyer, I.M. , Bhardwaj, A. , Sahakian, B.J. , Jones, P.B. , & Wilkinson, P.O. (2022). Behavioral measures of impulsivity and compulsivity in adolescents with nonsuicidal self‐injury. CNS Spectrums, 27, 604–612. 33888173 10.1017/S1092852921000274 PMC7613746

[jcpp70018-bib-0051] McGlinchey, E.L. , Courtney‐Seidler, E.A. , German, M. , & Miller, A.L. (2017). The role of sleep disturbance in suicidal and nonsuicidal self‐injurious behavior among adolescents. Suicide and Life‐threatening Behavior, 47, 103–111. 27273654 10.1111/sltb.12268

[jcpp70018-bib-0052] McHugh, C.M. , Chun Lee, R.S. , Hermens, D.F. , Corderoy, A. , Large, M. , & Hickie, I.B. (2019). Impulsivity in the self‐harm and suicidal behavior of young people: A systematic review and meta‐analysis. Journal of Psychiatric Research, 116, 51–60. 31195164 10.1016/j.jpsychires.2019.05.012

[jcpp70018-bib-0053] Medrzycka‐Dabrowska, W. , Lewandowska, K. , Kwiecień‐Jaguś, K. , & Czyż‐Szypenbajl, K. (2018). Sleep deprivation in intensive care unit – Systematic review. Open Medicine, 13, 384–393. 30211321 10.1515/med-2018-0057 PMC6132084

[jcpp70018-bib-0054] Meltzer, L.J. , Wainer, A. , Engstrom, E. , Pepa, L. , & Mindell, J.A. (2021). Seeing the whole elephant: A scoping review of behavioral treatments for pediatric insomnia. Sleep Medicine Reviews, 56, 101410. 33387973 10.1016/j.smrv.2020.101410

[jcpp70018-bib-0055] Metten, M.‐A. , Costet, N. , Multigner, L. , Viel, J.‐F. , & Chauvet, G. (2022). Inverse probability weighting to handle attrition in cohort studies: Some guidance and a call for caution. BMC Medical Research Methodology, 22, 45. 35172753 10.1186/s12874-022-01533-9 PMC8848672

[jcpp70018-bib-0056] Midi, H. , Sarkar, S.K. , & Rana, S. (2010). Collinearity diagnostics of binary logistic regression model. Journal of Interdisciplinary Mathematics, 13, 253–267.

[jcpp70018-bib-0057] Moller, C.I. , Tait, R.J. , & Byrne, D.G. (2013). Deliberate self‐harm, substance use, and negative affect in nonclinical samples: A systematic review. Substance Abuse, 34, 188–207. 23577914 10.1080/08897077.2012.693462

[jcpp70018-bib-0058] Morin, C.M. , Rodrigue, S. , & Ivers, H. (2003). Role of stress, arousal, and coping skills in primary insomnia. Psychosomatic Medicine, 65, 259–267. 12651993 10.1097/01.psy.0000030391.09558.a3

[jcpp70018-bib-0059] Nguyen, T.P. , Lerch, S. , Maggetti, A. , Reichl, C. , Tarokh, L. , & Kaess, M. (2023). The relationship between sleep disturbance and self‐harming behaviours in high‐risk clinical adolescents. Journal of Psychiatric Research, 158, 81–87. 36577237 10.1016/j.jpsychires.2022.12.034

[jcpp70018-bib-0060] Ohayon, M.M. , Krystal, A. , Roehrs, T.A. , Roth, T. , & Vitiello, M.V. (2010). Using difficulty resuming sleep to define nocturnal awakenings. Sleep Medicine, 11, 236–241. 20075004 10.1016/j.sleep.2009.11.004 PMC2830306

[jcpp70018-bib-0061] Oktan, V. (2017). Self‐harm behaviour in adolescents: Body image and self‐esteem. Journal of Psychologists and Counsellors in Schools, 27, 177–189.

[jcpp70018-bib-0062] Oldershaw, A. , Grima, E. , Jollant, F. , Richards, C. , Simic, M. , Taylor, L. , & Schmidt, U. (2009). Decision making and problem solving in adolescents who deliberately self‐harm. Psychological Medicine, 39, 95–104. 18570698 10.1017/S0033291708003693

[jcpp70018-bib-0063] Patte, K.A. , Qian, W. , & Leatherdale, S.T. (2017). Sleep duration trends and trajectories among youth in the COMPASS study. Sleep Health, 3, 309–316. 28923185 10.1016/j.sleh.2017.06.006

[jcpp70018-bib-0064] Perlis, M. , Shaw, P.J. , Cano, G. , & Espie, C.A. (2011). Models of insomnia. In M.H. Kryger , R. Thomas & W.C. Dement (Eds.), Principles and practice of sleep medicine (pp. 850–865). Philadelphia, PA: Saunders/Elsevier.

[jcpp70018-bib-0065] Pezzi, A. , Cavo, M. , Biggeri, A. , Zamagni, E. , & Nanni, O. (2016). Inverse probability weighting to estimate causal effect of a singular phase in a multiphase randomized clinical trial for multiple myeloma. BMC Medical Research Methodology, 16, 150. 27829371 10.1186/s12874-016-0253-9 PMC5103416

[jcpp70018-bib-0066] Pitkänen, J. , Bijlsma, M.J. , Remes, H. , Aaltonen, M. , & Martikainen, P. (2021). The effect of low childhood income on self‐harm in young adulthood: Mediation by adolescent mental health, behavioural factors and school performance. SSM – Population Health, 13, 100756. 33681447 10.1016/j.ssmph.2021.100756 PMC7910518

[jcpp70018-bib-0069] Riemann, D. , Espie, C.A. , Altena, E. , Arnardottir, E.S. , Baglioni, C. , Bassetti, C.L.A. , … & Spiegelhalder, K. (2023). The European insomnia guideline: An update on the diagnosis and treatment of insomnia 2023. Journal of Sleep Research, 32, e14035. 38016484 10.1111/jsr.14035

[jcpp70018-bib-0068] Riemann, D. , Spiegelhalder, K. , Nissen, C. , Hirscher, V. , Baglioni, C. , & Feige, B. (2012). REM sleep instability – A new pathway for insomnia? Pharmacopsychiatry, 45, 167–176. 22290199 10.1055/s-0031-1299721

[jcpp70018-bib-0070] Rogers, R.D. (1999). Dissociable deficits in the decision‐making cognition of chronic amphetamine abusers, opiate abusers, patients with focal damage to prefrontal cortex, and tryptophan‐depleted normal volunteers evidence for monoaminergic mechanisms. Neuropsychopharmacology, 20, 322–339. 10088133 10.1016/S0893-133X(98)00091-8

[jcpp70018-bib-0071] Romeu, R.J. , Haines, N. , Ahn, W.‐Y. , Busemeyer, J.R. , & Vassileva, J. (2020). A computational model of the Cambridge Gambling task with applications to substance use disorders. Drug and Alcohol Dependence, 206, 107711. 31735532 10.1016/j.drugalcdep.2019.107711 PMC6980771

[jcpp70018-bib-0072] Rosales‐Lagarde, A. , Armony, J.L. , del Río‐Portilla, Y. , Trejo‐Martínez, D. , Conde, R. , & Corsi‐Cabrera, M. (2012). Enhanced emotional reactivity after selective REM sleep deprivation in humans: An fMRI study. Frontiers in Behavioral Neuroscience, 6. 10.3389/fnbeh.2012.00025 PMC3376727 22719723

[jcpp70018-bib-0073] Rosenberg, M. (1965). Society and the adolescent self‐image. Princeton: Princeton University Press.

[jcpp70018-bib-0074] Russell, K. , Allan, S. , Beattie, L. , Bohan, J. , MacMahon, K. , & Rasmussen, S. (2019). Sleep problem, suicide and self‐harm in university students: A systematic review. Sleep Medicine Reviews, 44, 58–69. 30721844 10.1016/j.smrv.2018.12.008

[jcpp70018-bib-0075] Satterfield, B.C. , & Killgore, W.D.S. (2019). Sleep loss, executive function, and decision‐making. In M.A. Gradner (Ed.), Sleep and health (pp. 339–358). London/San Diego/Cambridge/Oxford: Elsevier.

[jcpp70018-bib-0076] Schmidt, S.C.E. , & Woll, A. (2017). Longitudinal drop‐out and weighting against its bias. BMC Medical Research Methodology, 17, 447–468. 10.1186/s12874-017-0446-x PMC5723086 29221434

[jcpp70018-bib-0077] Scott, H. , Biello, S.M. , & Woods, H.C. (2019). Social media use and adolescent sleep patterns: Cross‐sectional findings from the UK millennium cohort study. BMJ Open, 9, e031161. 10.1136/bmjopen-2019-031161 PMC6830469 31641035

[jcpp70018-bib-0078] Seaman, S.R. , & White, I.R. (2013). Review of inverse probability weighting for dealing with missing data. Statistical Methods in Medical Research, 22, 278–295. 21220355 10.1177/0962280210395740

[jcpp70018-bib-0079] Seton, C. , & Fitzgerald, D.A. (2021). Chronic sleep deprivation in teenagers: Practical ways to help. Paediatric Respiratory Reviews, 40, 73–79. 34144910 10.1016/j.prrv.2021.05.001

[jcpp70018-bib-0080] Shepherd, P. , & Gilbert, E. (2019). Millennium Cohort Study: Ethical review and consent. London: Centre for Longitudinal Studies.

[jcpp70018-bib-0081] Su, R. , John, J.R. , & Lin, P.‐I. (2023). Machine learning‐based prediction for self‐harm and suicide attempts in adolescents. Psychiatry Research, 328, 115446. 37683319 10.1016/j.psychres.2023.115446

[jcpp70018-bib-0082] Torun, Y. , Gul, H. , Sahin, M. , Hekim, O. , Ates, B. , Bodur, S. , … & Congologlu, M. (2021). Daydreaming and sluggishness may predict different functions for non‐suicidal self‐injury: A clinic‐based single‐center study. Psychiatry and Behavioral Sciences, 11, 267.

[jcpp70018-bib-0083] Trouche, S. , Pompili, M.N. , & Girardeau, G. (2020). The role of sleep in emotional processing: Insights and unknowns from rodent research. Current Opinion in Physiology, 15, 230–237.

[jcpp70018-bib-0084] Venkatraman, V. , Chuah, Y.L. , Huettel, S.A. , & Chee, M.W. (2007). Sleep deprivation elevates expectation of gains and attenuates response to losses following risky decisions. Sleep, 30, 603–609. 17552375 10.1093/sleep/30.5.603

[jcpp70018-bib-0085] Vuong, J.S. , York, A. , Garrett, J. , Connolly, M.J. , & Devergnas, A. (2021). Sleep fragmentation and decreased REM sleep in a primate model of diurnal cortical seizures. Epilepsy Research, 178, 106805. 34768048 10.1016/j.eplepsyres.2021.106805 PMC12118802

[jcpp70018-bib-0086] Wang, Y. , Dai, C. , Shao, Y. , Wang, C. , & Zhou, Q. (2022). Changes in ventromedial prefrontal cortex functional connectivity are correlated with increased risk‐taking after total sleep deprivation. Behavioural Brain Research, 418, 113674. 34798167 10.1016/j.bbr.2021.113674

[jcpp70018-bib-0087] Wardle‐Pinkston, S. , Slavish, D.C. , & Taylor, D.J. (2019). Insomnia and cognitive performance: A systematic review and meta‐analysis. Sleep Medicine Reviews, 48, 101205. 31522135 10.1016/j.smrv.2019.07.008

[jcpp70018-bib-0088] Wassing, R. , Lakbila‐Kamal, O. , Ramautar, J.R. , Stoffers, D. , Schalkwijk, F. , & Van Someren, E.J.W. (2019). Restless REM sleep impedes overnight amygdala adaptation. Current Biology, 29, 2351–2358.e4. 31303489 10.1016/j.cub.2019.06.034

[jcpp70018-bib-0089] Wittmann, M. , Dinich, J. , Merrow, M. , & Roenneberg, T. (2006). Social jetlag: Misalignment of biological and social time. Chronobiology International, 23, 497–509. 16687322 10.1080/07420520500545979

[jcpp70018-bib-0090] Wood, W.L.M. , Lewandowski, L.J. , Lovett, B.J. , & Antshel, K.M. (2017). Executive dysfunction and functional impairment associated with sluggish cognitive tempo in emerging adulthood. Journal of Attention Disorders, 21, 691–700. 25520166 10.1177/1087054714560822

