# Supplementary material for: The longitudinal role of sleep on self‐harm during adolescence: A birth cohort study
Source: J Child Psychol Psychiatry. 2025 Aug 4;67(1):67–78. doi: 10.1111/jcpp.70018 (PMC12699108; doi:10.1111/jcpp.70018)
Supplement: Supplementary file 1 — Appendix S1. Supplementary material. Table S1. Associations between various variables possibly related to attrition and participation at 17 years of age. Table S2. Correlations between all sleep variables with rational decision‐making, depressive symptoms at 14, self‐esteem at 14 years. Table S3. Weighted unadjusted and adjusted interactions between sleep and rational decision‐making at 14 years with self‐harm at 17 years. Figure S1. Direct path diagram between sleep variables at 14 years, rational decision‐making at 14 years, and self‐harm at 17 years, with sex, ethnicity, prior self‐harm, self‐esteem, and depressive symptoms at 14 as control variables. [file JCPP-67-67-s001.docx]

**Supplementary Material**

**Methods**

**Measures**

***Measures of Self-Reported Sleep Variables:*** Some of the specific sleep items developed by the MCS team have been included, and occasionally slightly altered, within validated sleep questionnaires. For example, within the Brief Infant Sleep Questionnaire (Sadeh, 2004), bedtime, sleep onset latency, and frequency of night awakenings are similarly assessed with the following items: “What time do you usually put your child to bed at night?”, “How long does it usually take your child to fall asleep?”, “How many times does your child usually wake during the night?”. Furthermore, the Pittsburgh Sleep Quality Index (Buysse, Reynolds, Monk, Berman, & Kupfer, 1989) has items relating to bedtime, sleep onset latency, wake-up time, and frequency of night awakenings that are closely worded to those included within the MCS questionnaire (“During the past month, what time have you usually gone to bed at night?”, “During the past month, how long (in minutes) has it usually taken you to fall asleep each night?”, “During the past month, what time have you usually gotten up in the morning?”, “During the past month, how often have you had trouble sleeping because you wake up in the middle of the night or early morning?”).

***Sleep Duration*.** Participants were asked: “About what time do you usually go to sleep on a school night/on the nights when you do not have school the next day?” and “About what time do you usually wake up in the morning on a school day/on the days when you do not have school?”. All response choices were coded following prior research (Hisler, Twenge, & Krizan, 2020; Jackson & Testa, 2022). For bedtime, response choices were recoded as the following: “Before 9pm,” (8:30), “9-9:59pm,” (9:30), “10-10:59pm,” (10:30), “11-midnight,” (11:30), “After midnight” (12:30). Wake-up response choices for school days were coded as: “Before 6am,” (5:30), “6-6:59am,” (6:30), “7-7:59am” (7:30), “8-8:59am” (8:30) and “After 9am” (9:30). Wake-up response choices for non-school days were recoded as: “Before 8am” (7:30), “8-8:59am” (8:30), “9-9:59am” (9:30), “10-10:59am” (10:30), “11-11:59am” (11:30), and “After midday” (12:30). To then generate values for sleep duration, the difference was taken between the recoded values corresponding to the same time period (e.g., wake-up time on school night – bedtime on school night).

***Sleep Onset Latency*.** Categorical options were recoded as “0-15 minutes” (7.5 min), “16-30 minutes” (23 min), “31-45 minutes” (38 min), “46-60 minutes” (53 min), and “More than 60 minutes” (90 min). This method of recoding mirrors previous research utilising this measure (Hisler et al., 2020).

***Night Awakenings*.** Again, original categorical response choices were recoded as “1 = All of the time” (5), “2 = Most of the time” (4), “3 = A good bit of the time” (3), “4 = Some of the time” (2), “5 = A little of the time” (1), and “6 = None of the time” (0). This inversion of the original scale was done so higher scores reflected more interrupted sleep to be consistent with the other sleep measures.

***Decision-Making*:** The CGT was administered to the MCS cohort members on a computer screen in their homes during sweep six (age 14). A row of ten boxes displayed as either red or blue appear at the top of the screen for each trial. Participants were informed that a “token” was hidden behind one box and needed to select which colour box (red or blue) it is behind. The task initiated with participants holding 100 points, which they selected a proportion of (between 5-95%) to gamble on each trial. A circle in the centre of the screen showed an incrementally increasing or decreasing bet value which the participant then selected to bet. Based on their decision, and which colour box the token is hidden behind, these points were then subtracted from or added to their total score. The ratio of blue to red boxes differed pseudo-randomly during the task to measure the effect of statistical probability on the quality of decision making. Sampling participants’ gambling behaviours across various risk levels, the CGT offers outcome measures for risk-taking, decision time, risk adjustment, delay aversion, overall proportion bet, and rational decision-making.

***Ethnicity*.** Ethnicity was initially recorded as 11 categories: White (N = 15,277; 82.6%), Mixed (N = 553; 3.0%), Indian (N = 465; 2.5%), Pakistani (N = 898; 4.9%), Bangladeshi (N = 367; 2.0%), Other Asian (N = 126; 0.7%), Black Caribbean (N = 243; 1.3%), Black African (N = 381; 2.1%), Other Black (N = 42; 0.2%), Chinese (N = 31; 0.2%), and Other Ethnic group (N = 109; 0.6%).

**Missing Data.**

Adolescents lost to attrition were more frequently white males, with a lower family income, lower gestational age, lower birth weight, with their natural mother having a younger maternal age, and not having completed higher education or an equivalent qualification.

**Table S1.** Associations Between Various Variables Possibly Related to Attrition and Participation At 17 Years of Age.

| **Participation at 17 years** | | | |
| --- | --- | --- | --- |
|  | **β** | ***P* value** | **OR (95% CI)** |
| Birth weight | 0.076 | 0.002 | 1.079 (1.028, 1.134) |
| Labour complications | 0.057 | 0.076 | 1.059 (0.994, 1.128) |
| Ethnicity | -0.141 | <0.001 | 0.868 (0.804, 0.937) |
| Sex | -0.212 | <0.001 | 0.809 (0.764, 0.857) |
| Family income | -0.545 | <0.001 | 0.580 (0.546, 0.616) |
| Longstanding illness | -0.095 | 0.126 | 0.910 (0.806, 1.027) |
| Depressive symptoms at 14 years | 0.006 | 0.193 | 1.006 (0.997, 1.014) |
| Have close friends at 14 years | 0.037 | 0.792 | 1.038 (0.787, 1.368) |
| Mother education level (Higher education/Other) | 0.674 | <0.001 | 1.962 (1.837, 2.095) |
| Gestational age | 0.004 | <0.001 | 1.004 (1.001, 1.006) |
| Maternal age when born | 0.044 | <0.001 | 1.045 (1.039, 1.050) |

**Results**

**Table S2.** Correlations Between All Sleep Variables with Rational Decision-Making, Depressive Symptoms at 14, Self-Esteem at 14 Years.

|  | **Sleep Duration Hours, School Days** | | **Sleep Duration Hours, Non-School Days** | | **Social Jetlag** | | **Sleep Onset Latency** | | **Night Awakenings** | |
| --- | --- | --- | --- | --- | --- | --- | --- | --- | --- | --- |
|  | **r** | ***p*** | **r** | ***p*** | **r** | ***p*** | **r** | ***p*** | **r** | ***p*** |
| Rational Decision-Making, 14 years | 0.026^a^ | 0.007 | -0.013^a^ | 0.176 | -0.040^a^ | <0.001 | -0.088^a^ | 0.384 | -0.070^a^ | <0.001 |
| Depressive Symptoms, 14 years | -0.255^a^ | <0.001 | -0.066^a^ | <0.001 | 0.045^a^ | <0.001 | 0.301^b^ | <0.001 | 0.377^b^ | <0.001 |
| Self-Esteem, 14 years | 0.192^a^ | <0.001 | 0.038^a^ | <0.001 | -0.059^a^ | <0.001 | -0.204^a^ | <0.001 | -0.249^a^ | <0.001 |

^a^ Weak correlations: r < 0.3; ^b^ Moderate correlations: r ≥ 0.3.

**Table S3.** Weighted Unadjusted and Adjusted Interactions between Sleep and Rational Decision-Making at 14 years with Self-Harm at 17 years.

|  | **Model A** | | | **Model B** | | |
| --- | --- | --- | --- | --- | --- | --- |
|  | **β** | ***P* value** | **OR (95% CI)** | **β** | ***P* value** | **OR (95% CI)** |
| Sleep Duration School Day X RDM | -0.374 | 0.044 | 0.688 (0.478, 0.991) | -0.258 | 0.223 | 0.772 (0.510, 1.170) |
| Sleep Duration School Day | 0.056 | 0.732 | 1.058 (0.767, 1.460) | 0.137 | 0.465 | 1.147 (0.794, 1.659) |
| RDM | 3.229 | 0.043 | 25.244 (1.114, 572.126) | 2.270 | 0.212 | 9.682 (0.273, 343.526) |
| Sex | - | - | - | -0.242 | <0.001 | 0.785 (0.697, 0.884) |
| Family Income | - | - | - | 0.068 | 0.285 | 1.070 (0.945, 1.211) |
| Ethnicity | - | - | - | 0.393 | <0.001 | 1.482 (1.250, 1.755) |
| Regular Smoking, 14 years | - | - | - | -0.028 | 0.889 | 0.972 (0.654, 1.445) |
| Previous Self-Harm, 14 years | - | - | - | 0.921 | <0.001 | 2.511 (2.148, 2.937) |
| Self-Esteem, 14 years | - | - | - | -0.042 | <0.001 | 0.959 (0.936, 0.983) |
| Depressive Symptoms, 14 years | - | - | - | 0.066 | <0.001 | 1.068 (1.055, 1.081) |
| Sleep Onset Latency X RDM | 0.018 | 0.021 | 1.018 (1.003, 1.033) | 0.011 | 0.221 | 1.011 (0.994, 1.028) |
| Sleep Onset Latency | -0.003 | 0.629 | 0.997 (0.984, 1.010) | -0.005 | 0.503 | 0.995 (0.980, 1.010) |
| RDM | -0.562 | 0.079 | 0.570 (0.304, 1.068) | -0.287 | 0.415 | 0.751 (0.376, 1.497) |
| Sex | - | - | - | -0.243 | <0.001 | 0.785 (0.697, 0.884) |
| Family Income | - | - | - | 0.071 | 0.260 | 1.074 (0.949, 1.215) |
| Ethnicity | - | - | - | 0.384 | <0.001 | 1.468 (1.239, 1.740) |
| Regular Smoking, 14 years | - | - | - | -0.007 | 0.971 | 0.993 (0.669, 1.474) |
| Previous Self-Harm, 14 years | - | - | - | 0.916 | <0.001 | 2.500 (2.138, 2.924) |
| Self-Esteem, 14 years | - | - | - | -0.042 | <0.001 | 0.958 (0.935, 0.982) |
| Depressive Symptoms, 14 years | - | - | - | 0.064 | <0.001 | 1.066 (1.053, 1.080) |
| Night Awakenings X RDM | 0.217 | 0.114 | 1.243 (0.949, 1.626) | 0.136 | 0.385 | 1.146 (0.843, 1.558) |
| Night Awakenings | 0.103 | 0.398 | 1.109 (0.873, 1.407) | -0.010 | 0.940 | 0.990 (0.753, 1.301) |
| RDM | -0.171 | 0.587 | 0.843 (0.456, 1.559) | -0.123 | 0.718 | 0.885 (0.455, 1.721) |
| Sex | - | - | - | -0.228 | <0.001 | 0.796 (0.707, 0.897) |
| Family Income | - | - | - | 0.032 | 0.613 | 1.033 (0.911, 1.170) |
| Ethnicity | - | - | - | 0.413 | <0.001 | 1.512 (1.275, 1.793) |
| Regular Smoking, 14 years | - | - | - | 0.003 | 0.987 | 1.003 (0.675, 1.490) |
| Previous Self-Harm, 14 years | - | - | - | 0.907 | <0.001 | 2.478 (2.118, 2.889) |
| Self-Esteem, 14 years | - | - | - | -0.044 | <0.001 | 0.957 (0.934, 0.981) |
| Depressive Symptoms, 14 years | - | - | - | 0.060 | <0.001 | 1.062 (1.049, 1.076) |

Abbreviation: RDM, Rational Decision-Making.

**Mediation Analysis**

Sleep duration on school days, sleep onset latency, and night awakenings were included as predictors in separate mediation analyses, with rational decision-making as a mediator and subsequent self-harm as the outcome. Covariates that were significantly associated with longitudinal self-harm in the adjusted model were also included (sex, ethnicity, prior self-harm, self-esteem and depressive symptoms at 14 years). These variables were included as control variables to account for their potential influence on the outcome. We used bootstrapped bias-corrected 95% confidence intervals and *P* values to assess the significance of the standardised direct and indirect effects. A 2-sided *P* value <0.05 was considered to be statistically significant.

In examining whether rational decision-making mediated the association between sleep duration on school days and subsequent self-harm, the path analysis model fit indices suggested good model fit (χ^2^ = 42.240, *p* <0.001, root mean square error of approximation 0.031, comparative fit index 0.996). Despite the chi-square indices being significant, which would suggest poor model fit, this assessment of global fit is sensitive to large sample sizes (Brannick, 1995). As the other model fit indices indicated excellent model fit, the analysis was continued. Direct associations are displayed in Figure S1A. We found an insignificant indirect effect of rational decision-making between exposure and outcome (bias-corrected estimate 0.000, 95% CI 0.000-0.001, *p* = 0.458).

With rational decision-making as a mediator of the association between sleep onset latency and longitudinal self-harm, model fit indexes indicated good model fit (χ^2^ =43.930, *p* <0.001, root mean square error of approximation 0.032, comparative fit index 0.995). Figure S1B illustrates the direct associations. However, no significant indirect effect of decision-making between sleep onset latency and self-harm was observed (bias-corrected estimate 0.000, 95% CI 0.000-0.000, *p* = 0.645).

For night awakenings as a predictor in the mediation analysis with decision-making and self-harm, the path analysis model was evaluated according to several structural equation modelling fit statistics and indices: χ^2^ = 34.158, *p* <0.001, root mean square error of approximation 0.028, comparative fit index 0.997. Direct associations are displayed in Figure S1C. The indirect effect of night awakenings on self-harm through rational decision-making was not significant (bias-corrected estimate 0.000, 95% CI −0.002 to 0.001, *p* = 0.533).

**Figure S1.** Direct Path Diagram Between Sleep Variables at 14 Years, Rational Decision-Making at 14 Years, and Self-Harm at 17 Years, with Sex, Ethnicity, Prior Self-Harm, Self-Esteem, and Depressive Symptoms at 14 as Control Variables.

A

β=-0.036, P=0.005

β=0.025, P=0.026

β=0.005, P=0.606

Sleep Duration School Day

Rational Decision-Making

Self-Harm

β=-0.003, P=0.794

β=0.004, P=0.701

B

Sleep Onset Latency

Rational Decision-Making

Self-Harm

β=0.042, P=<0.001

β=-0.054, P<0.001

β=0.007, P=0.575

C

Night Awakenings

Rational Decision-Making

Self-Harm

β=0.055, P<0.001

**References**

Brannick, M. T. (1995). Critical comments on applying covariance structure modeling. *Journal of Organizational Behavior*, *16*(3), 201–213.

Buysse, D. J., Reynolds, C. F., Monk, T. H., Berman, S. R., & Kupfer, D. J. (1989). The Pittsburgh Sleep Quality Index: a new instrument for psychiatric practice and research. *Psychiatry research*, *28*(2), 193–213.

Hisler, G., Twenge, J. M., & Krizan, Z. (2020). Associations between screen time and short sleep duration among adolescents varies by media type: evidence from a cohort study. *Sleep Medicine*, *66*, 92–102.

Jackson, D. B., & Testa, A. (2022). Police stops and adolescent sleep problems: findings from the UK millennium cohort study. *Journal of Sleep Research*, *31*(5).

Sadeh, A. (2004). A Brief Screening Questionnaire for Infant Sleep Problems: Validation and Findings for an Internet Sample. *Pediatrics*, *113*(6), e570–e577.
